# Regulatory Potential of piRNAs Targeting *Klotho* and Other Genes

**DOI:** 10.3390/genes17020241

**Published:** 2026-02-18

**Authors:** Anna Pyrkova, Kyrmyzy Akhmetova, Murat Zhanuzakov, Makpal Tauassarova, Aizhan Rakhmetulina, Raigul Niyazova, Saltanat Orazova, Piotr Zielenkiewicz, Anatoliy Ivashchenko

**Affiliations:** 1 Department of Computer Sciences, Al-Farabi Kazakh National University, Almaty 050040, Kazakhstan; 2 Higher School of Medicine, Al-Farabi Kazakh National University, Almaty 050040, Kazakhstan; 3 Institute of Biochemistry and Biophysics, Polish Academy of Sciences, 02-106 Warsaw, Poland; 4 Department of Biotechnology, Al-Farabi Kazakh National University, Almaty 050040, Kazakhstan; 5 Institute of Experimental Plant Biology and Biotechnology, University of Warsaw, 02-096 Warsaw, Poland; 6 Center for Bioinformatics and Nanomedicine, Almaty 050060, Kazakhstan

**Keywords:** piRNAs, miRNA, mRNA, *Klotho* gene, human, aging, disease

## Abstract

Background/Objectives: piRNAs (PIWI-interacting RNAs) can significantly modify the expression of protein-coding genes by suppressing the translation process. The aim of this work was to computationally evaluate the potential interactions between piRNAs and the mRNA of the *Klotho* gene, as well as other genes involved in key metabolic pathways related to health and lifespan regulation. Methods: Bioinformatic analysis was conducted using the MirTarget program, which determines the quantitative characteristics of predicted nucleotide interactions between piRNAs and mRNA targets. Results: Several piRNAs (piR-44682, piR-1940042, piR-3008660, piR-3215034, piR-6885965, and piR-7980636) were predicted to bind within a single cluster of binding sites on the *KL* mRNA. In addition, piR-6890096 was predicted to interact with the *KL* mRNA through full complementarity. The mRNAs of *AFF2*, *BCL2L11*, *CPT1A*, *DAZAP1*, *NDRG3*, *SKIDA1*, *WBP4*, *ZIC5*, and *ZSWIM6* were predicted to interact with piR-3215034 and piR-6885965, forming clusters of binding sites located in the 5′ untranslated region (5′UTR), coding sequence (CDS), and 3′ untranslated region (3′UTR). Additionally, piR-576442, piR-1501557, piR-1845735, piR-2069834, and piR-3029987 were predicted to bind only within the 3′UTR of *FGF23* mRNA. These results suggest that piRNAs are potential regulators of *KL* and other genes involved in key metabolic processes. Conclusions: The findings provide a basis for further experimental validation of predicted piRNA–mRNA interactions and their possible roles in gene regulation.

## 1. Introduction

The *Klotho* (*KL*) gene belongs to a family consisting of α-klotho, β-klotho, and γ-klotho. In the present study, we focus on the α-klotho subfamily, hereafter referred to as Klotho. The *KL* gene is highly expressed in the brain, liver, and kidney, and has attracted substantial interest among biologists and physicians due to its involvement in diverse metabolic pathways and disease processes. Notably, reduced Klotho protein levels are associated with increased mortality in various diseases. Its molecular, physiological, and therapeutic characteristics have been extensively reviewed [1]. Growing evidence highlights the diagnostic and therapeutic potential of *KL* [2]. In particular, low serum Klotho levels serve as a reliable biomarker for both the severity of cardiovascular-renal-metabolic disorders and the corresponding risk of mortality [3]. Consequently, exogenous Klotho supplementation has emerged as a promising therapeutic approach. Future investigations may clarify its utility in diabetic nephropathy and other diseases [4]. Recent research has also explored epigenetic regulation of *KL* expression in various diseases [5,6], providing deeper insight into the mechanisms controlling its activity. The role of *KL* in diabetes has been increasingly recognized [7,8,9,10,11], and numerous studies have examined its function in kidney diseases [12,13,14]. Additionally, accumulating evidence supports the involvement of Klotho in cancer biology [15,16,17,18,19]. A substantial body of work has also addressed the relationship between *KL* and Fibroblast Growth Factor 23 (*FGF23*) [20,21,22,23]. Beyond disease-specific roles, the *KL* gene has gained particular attention as a key regulator of aging [24,25,26]. Collectively, these findings underscore the broad biological significance of *KL* and its potential as a therapeutic target.

miRNAs (mRNA-inhibiting RNA), small non-coding RNAs that inhibit mRNA translation, play a critical role in the post-transcriptional regulation of gene expression. Consequently, identifying miRNAs that influence *KL* gene expression is essential for understanding the molecular mechanisms underlying its dysregulation in disease. Several studies have reported that specific miRNAs modulate Klotho protein levels. For example, miR-34a suppresses *KL* expression by directly binding to the 3′-untranslated region (3′-UTR) of its mRNA [27]. miR-339 has also been implicated in the regulation of *KL* expression, potentially contributing to altered gene expression patterns observed in patients with schizophrenia.

Recent studies have therefore focused on elucidating the role of miR-339-5p in controlling *KL* gene expression [28,29]. In addition, the Klotho-derived peptide KP1 has been shown to inhibit cellular senescence by restoring endogenous *KL* expression, and miR-556-3p was identified as an indirect repressor of the *KL* gene [30]. miR-200c has been demonstrated to modulate *KL* expression in human kidney cells exposed to oxidative stress [31], whereas miR-379 regulates *KL* synthesis in the context of apoptosis [32]. Furthermore, KP1 peptide-induced suppression of cellular senescence has been linked to enhanced *KL* expression via post-transcriptional regulation mediated by miR-223-3p [33]. This list of miRNAs associated with *KL* regulation is not exhaustive and requires further verification. In the present study, we analyzed the interactions between known Klotho-related miRNAs and *KL* mRNA to clarify their regulatory roles.

Compared with miRNAs, piRNAs (PIWI-interacting RNAs) have been far less studied in the context of *KL* mRNA regulation. Like miRNAs, piRNAs are thought to regulate the expression of protein-coding genes, primarily through post-transcriptional mechanisms [34,35]. Therefore, identifying which piRNAs can directly bind to *KL* mRNA is an important step toward understanding their potential regulatory roles. piRNAs are capable of substantially altering gene expression by suppressing translation, and because they function as endogenous regulators, they hold promise for both diagnostic and therapeutic applications. However, their investigation is complicated by the extremely large number of known piRNAs [36]. Without computational approaches, determining which piRNAs may target *KL*—or other genes—is highly challenging. Furthermore, it is important to assess whether piRNAs and miRNAs that interact with *KL* mRNA also target additional genes, thereby potentially causing off-target effects. For these reasons, effective computational tools are essential for studying the interactions of piRNAs and miRNAs with their mRNA targets. In this study, we employed the MirTarget program to analyze the quantitative characteristics of piRNA with the mRNA of target genes. This program has demonstrated reliability and effectiveness in previous studies.

## 2. Materials and Methods

The nucleotide (nt) sequence of *KL* gene and other genes were downloaded from National Center for Biotechnology Information database NCBI (https://www.ncbi.nlm.nih.gov, 2022). The nucleotide sequences of 8,480,000 piRNAs were taken from Wang et al. publication [36]. The 3707 miRNAs were taken from Londin et al. article [37], the 2567 mature miRNA sequences were taken from the miRBase database (http://mirbase.org), and the 1036 miRNAs from the article by Backes et al. [38].

The piRNA and miRNA binding sites (BSs) in mRNA were predicted using the MirTarget program [39]. This program predicts the following features of piRNA and miRNA binding to mRNA: (a) the initiation of piRNA and miRNA binding to the mRNA from the first nucleotide of the mRNA; (b) the localization of the piRNA and miRNA BSs in the 5′-untranslated region (5′UTR), coding domain sequence (CDS), and 3′-untranslated region (3′UTR) of the mRNAs; (c) the schemes of nucleotide interactions between piRNAs and miRNA with mRNA; (d) the free energy of the interaction between piRNAs, miRNA and the mRNA (ΔG, kJ/mol); and the ratio ΔG/ΔGm (%) is determined for each site. ΔGm equals the free energy of piRNA and miRNA binding with its fully complementary canonical nucleotide sequence. Only piRNAs and miRNAs whose nucleotides interacted with mRNA using canonical (G-C and A-U) and noncanonical (G-U and A-C) nucleotides with a given ΔG value were selected from the calculated data [40,41]. The MirTarget program finds hydrogen bonds between piRNAs and miRNAs with mRNA according to the physicochemical characteristics of nucleotide interactions.

MirTarget differs from other programs in terms of finding piRNA BSs on mRNA in the following: it takes into account the interaction of piRNA and miRNA with mRNA over the entire piRNA and miRNA nucleotides sequence; it considers noncanonical pairs G–U and A–C; and it calculates the free energy of the interaction of the piRNAs and miRNA with mRNA. Note that the G, A, C, and U nucleotides, which comprise the RNA structure of microorganisms, plants, and animals, interact identically under equal conditions. Therefore, the physicochemical properties of canonical and noncanonical nucleotide pairs given above do not require additional proof of the previously established physicochemical characteristics of their interaction. The reliability of translation suppression by miRNAs that are fully complementary to mRNAs was proven by A. Fire and C.C. Mello, who were awarded the Nobel Prize in 2006 for this research [42].

## 3. Results

**Figure 1:**
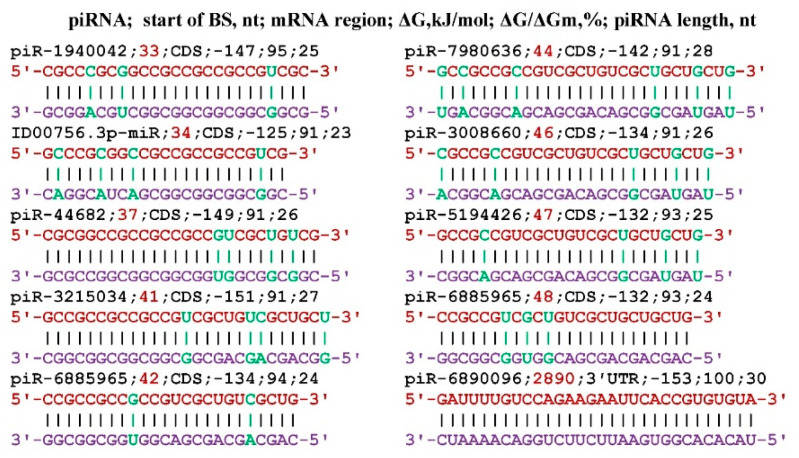
Schemes of the interaction of piRNA and miRNA with mRNA of the *Klotho* gene. Note: the mRNA nucleotides are highlighted in red. The piRNA nucleotides that form canonical pairs with mRNA are highlighted in violet and noncanonical pairs are highlighted in green.

Of the entire piRNA database, only seven piRNAs (piR-44682, piR-1940042, piR-3008660, piR-3215034, piR-5194426, piR-6885965, piR-7980636) bound to the mRNA of *KL* gene (Figure 1). Seven piRNAs each had one BSs and piR-6885965 had two BSs. All BSs were located with a partial overlap of nucleotides, forming a cluster of BSs from 33 nt to 71 nt, only 39 nt long. Such an arrangement of BSs piRNAs in CDS mRNA leads to competition between them for binding to mRNA. piR-6890096 interacts with 3′UTR in a completely complementary manner (the value of ΔG/ΔGm is 100%), which, at its concentration comparable to that of mRNA, will lead to inhibition of the translation process.

Note that the term miRNA or piRNA binding cluster introduced by us differs from the term cluster of the location of several genes encoding miRNA in a section of the chromosome. The *KL* gene is many times more strongly expressed in the kidneys than in other organs, and a high concentration of piRNA is required to suppress its synthesis. Of the 7310 miRNAs, only ID00756.3p-miR is bound to the mRNA of the *KL* gene (Figure 1). ID00756.3p-miR can only interact with mRNA of the *KCNN2*, *NOTCH3*, and *ZNF592* genes. The ID00756.3p-miR was in the BSs cluster of seven piRNAs located in the CDS mRNA *KL* gene; therefore, it competed with them.

Out of 8,405,000 piRNAs, only eight piRNAs were identified, of which piRNA-6885965 has two BSs in the mRNA of the *KL* gene. piRNA-6890096 interacted with the mRNA of the *KL* gene in a completely complementary manner using only canonical nucleotides. Out of 7310 miRNAs, only ID00756.3p-miR could bind to the mRNA of the *KL* gene. The piRNA (except piRNA-6890096) and miRNA bound in a 39 nt region (Figure 1). Here and henceforth, piRNA BSs with overlapping nucleotide sequences will be referred to as BSs clusters. As a result of this arrangement, piRNA BSs was compete each other for interaction with the target gene mRNA, and as a result, only one piRNA that binds mRNA more strongly than others or is in a significantly higher concentration will be bound for a longer time than other piRNAs. The formation of clusters of piRNA and miRNA BSs in mRNA is a kind of guarantee of the non-random association of small RNAs and their target genes.

The results obtained give reason for the possibility of specific regulation of *KL* gene expression using piRNAs and miRNAs. However, given that some miRNAs and piRNAs can bind to mRNAs of several or even hundreds of human genes [43], it is necessary to identify human genes that may be affected by piRNAs and miRNAs that act on mRNAs of the *KL* gene. That is, it is necessary to identify the possible side effect of these piRNA and miRNA on the expression of all human genes if they are used as therapeutic drugs. To this end, we studied the possible binding of ID00756.3p-miR and nine piRNAs to 17,484 human genes.

**Figure 2:**
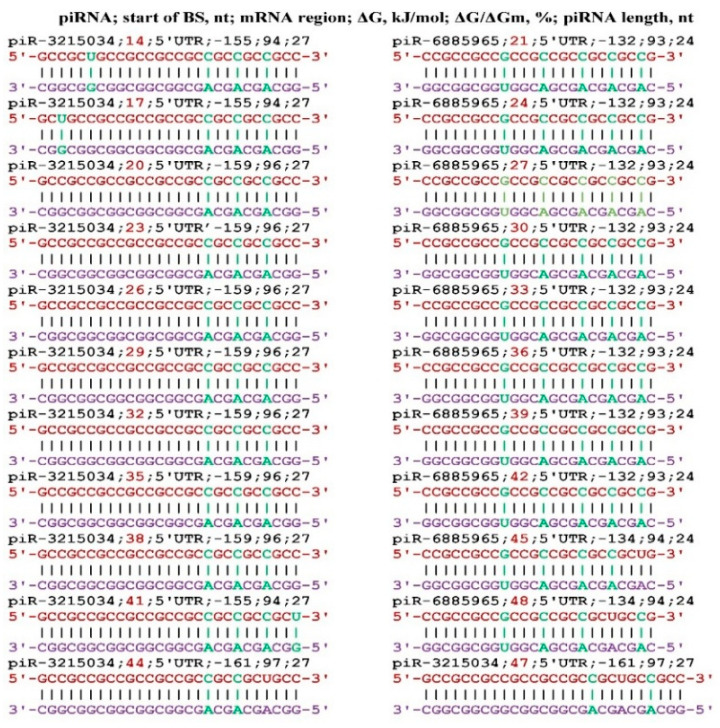
Schemes of the interaction of piR-3215034 and piR-6885965 with the mRNA of the *AFF2* gene.

The largest number of target genes was found for piR-3215034 and piR-6885965. In the mRNA of the *AFF2* gene, for each of these piRNAs, eleven and ten BSs were found, respectively, located in one cluster (Figure 2). All BSs was located the 5′UTR through three nucleotides. The free energy of piR-3215034 binding to the mRNA of the *AFF2* gene varied from −155 kJ/mol to −161 kJ/mol, and the ΔG/ΔGm value varied from 94% to 97%. During the piR-3215034 interaction with mRNA, canonical nucleotide pairs was involved in the last two BSs, except for two C-A bonds. These results indicate a high efficiency of piR-3215034 influence on *AFF2* gene expression. piR-6885965 is 24 nts long and, therefore, the free energy of interaction with the mRNA of the *AFF2* gene was lower. Since the BSs of these piRNAs are in the same cluster, piR-3215034 has the advantage of binding. However, at a significantly higher concentration of piR-6885965, it will have an advantage in binding over piR-3215034. Therefore, when determining the effectiveness of the action of piRNAs on translation, one should take into account the free energy of their interaction with mRNA and the concentration of competing piRNAs.

Since the *AFF2* and *KL* genes are targets for piR-3215034 and piR-6885965, it is necessary to compare the possible effect of piRNAs on these genes. It should be noted that the *AFF2* gene is involved in the development of squamous cell carcinoma [44], thoracic carcinoma [45], carcinomas of head and neck [46], and renal cell carcinoma and other diseases [47,48,49]. Therefore, by suppressing the oncogenesis caused by the *AFF2* gene with piR-3215034 and piR-6885965, the expression of *KL* gene will be simultaneously suppressed. No other piRNAs interacting with mRNA of the *AFF2* gene were identified. These results clearly demonstrate that, for each miRNA or piRNA, it is necessary to determine the expression of the specific genes they may influence.

**Figure 3:**
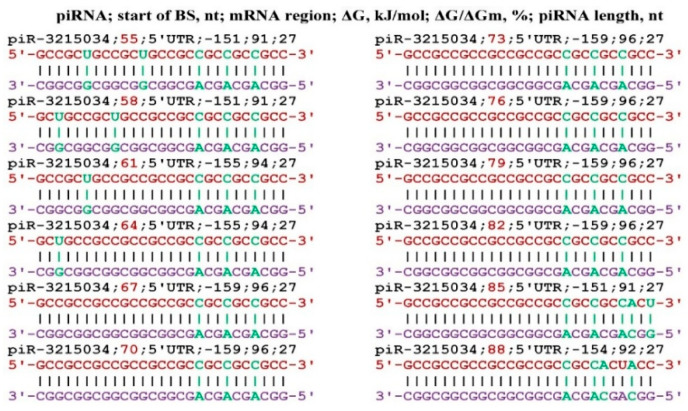
Schemes of the interaction of piR-3215034 with the mRNA of the *BCL2L11* gene.

Several publications have established the involvement of the *BCL2L11* gene in oncogenesis [50,51]. For piR-3215034, 12 BSs were identified, which form a cluster of BSs from 55 nt to 114 nt, 60 nt long (Figure 3). piR-6885965 also binds in this BSs cluster, but with a ΔG/ΔGm value of less than 90%, which is below the selection criterion for significant piRNAs. No other piRNAs interacting with mRNA of the *AFF2* gene were identified.

**Figure 4:**
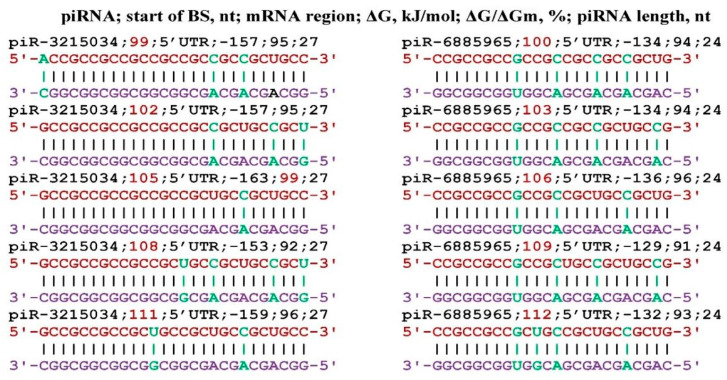
Interaction schemes of piR-3215034 and piR-6885965 with mRNA of the *CPT1A* gene.

The *CPT1A* gene is involved in fatty acid metabolism and manifests its effect during oncogenesis, diabetes, and cardiomyopathy [52,53,54,55,56,57]. Figure 4 shows the interaction schemes of piR-3215034 and piR-6885965 with the mRNA of the *CPT1A* gene, which show that their BSs are located in the same cluster from 99 nt to 137 nt. piR-3215034 interacts with the mRNA of the *CPT1A* gene with a ΔG/ΔGm value of 99%, i.e., almost canonical base pairs are formed. No other piRNAs interacting with mRNA of the *CPT1A* gene were identified.

**Figure 5:**
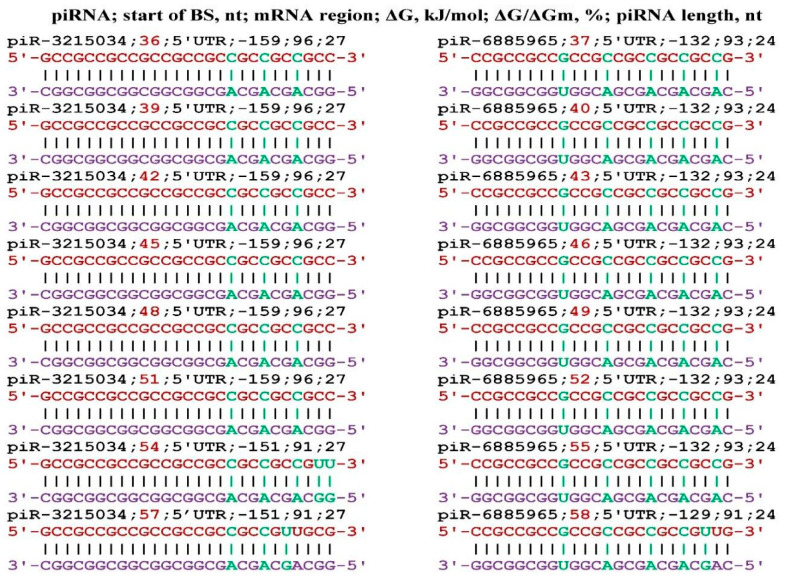
Schemes of the interaction of piR-3215034 and piR-6885965 with mRNA of the *DAZAP1* gene.

High expression of the *DAZAP1* gene is observed in hepatocarcinoma and can serve as a prognostic marker of the disease. Knockdown of *DAZAP1* small interfering RNA markedly inhibited proliferation, migration, and invasion of hepatocarcinoma cells [58,59]. piR-3215034 and piR-6885965 can repress *DAZAP1* mRNA translation (Figure 5).

The results obtained indicate that these piRNAs can significantly influence the expression of the *DAZAP1* gene (Figure 5). It should be noted that both piRNAs bind to the mRNA of the gene in the same cluster of BSs located in the 5′UTR, i.e., they can stop protein synthesis before the translation process. The *DAZAP1* gene is highly expressed in the testis (RPKM 19.6), and appendix (RPKM 11.8). No other piRNAs interacting with mRNA of the *DAZAP1* gene were identified.

A number of publications have shown the involvement of the *NDRG3* gene in the development of oncogenesis, and in most cases, increased expression is observed in cancer of various organs [60,61,62,63,64,65]. Therefore, it is important to know whether piR-3215034 and piR-6885965 can suppress the expression of the *NDRG3* gene.

**Figure 6:**
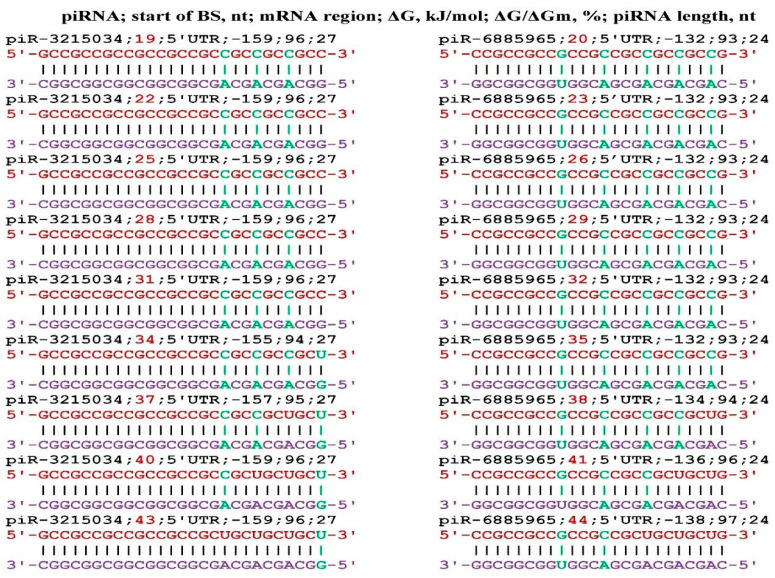
Schemes of the interaction of piR-3215034 and piR-6885965 with mRNA of the *NDRG3* gene.

No other piRNAs interacting with mRNA of the *NDRG3* gene were identified. The results shown in Figure 6 indicate that *NDRG3* gene expression can be downregulated by piR-3215034 and piR-6885965. No other piRNAs interacting with mRNA of the *NDRG3* gene were identified.

**Figure 7:**
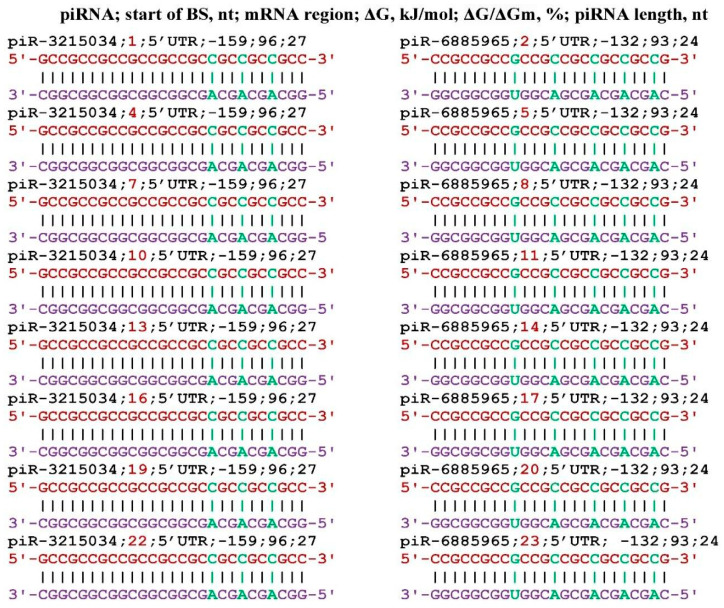
Interaction schemes of piR-3215034 and piR-6885965 with mRNA of the *RHOT1* gene.

The *RHOT1* gene is involved in the modification of the development of breast cancer [66], pancreatic cancer [67,68], non-small cell lung cancer, hepatocellular carcinoma [69], and the risk and occurrence of Parkinson’s disease [70]. Figure 7 shows the interaction schemes of piR-3215034 and piR-6885965 with mRNA of the *RHOT1* gene. Both piRNAs have eight BSs in the 5′UTR of the mRNA *RHOT1* gene located in the same BSs cluster from the first nucleotide to 48 nt. The next BS was located after three nucleotides, and the free energy of interaction was the same in each of the sites for both piRNAs. Therefore, the value of ΔG/ΔGm was the same for each piRNA. No other piRNAs interacting with mRNA of the *RHOT1* gene were identified.

**Figure 8:**
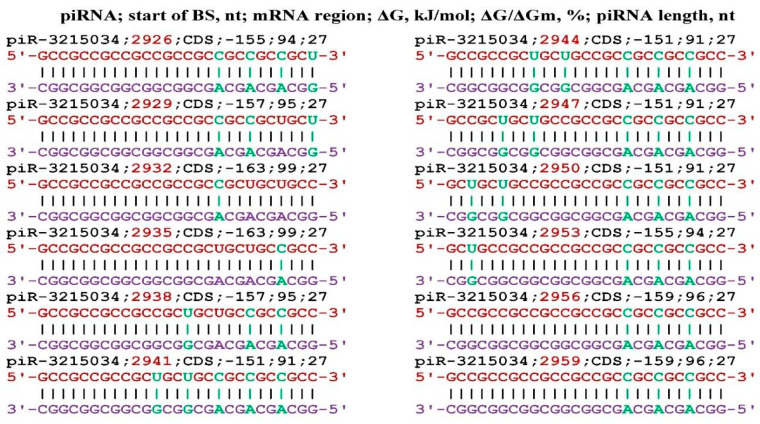
Schemes of the interaction of piR-3215034 with the mRNA of the *SKIDA1* gene.

*SKIDA1* was significantly overexpressed in all molecular subgroups, except for only two subgroups of acute myeloid leukemia. In validation analyses, *SKIDA1* was associated with higher sensitivity and specificity in acute myeloid leukemia. We highlight that *SKIDA1* is one of the promising markers, which has consistent overexpression among several types of acute leukemia [71,72]. *SKIDA1* associated with obesity [73]. For piR-3215034, 12 BSs were identified in the mRNA *SKIDA1* gene (Figure 8). At two sites, piR-3215034 interacts with mRNA almost as complementary as possible, since the ΔG/ΔGm value is 99%. A cluster of piR-3215034 BSs with 61 nt in length guarantees the binding of two of these 27 nt piRNAs at once. No other piRNAs interacting with mRNA of the *SKIDA1* gene were identified.

**Figure 9:**
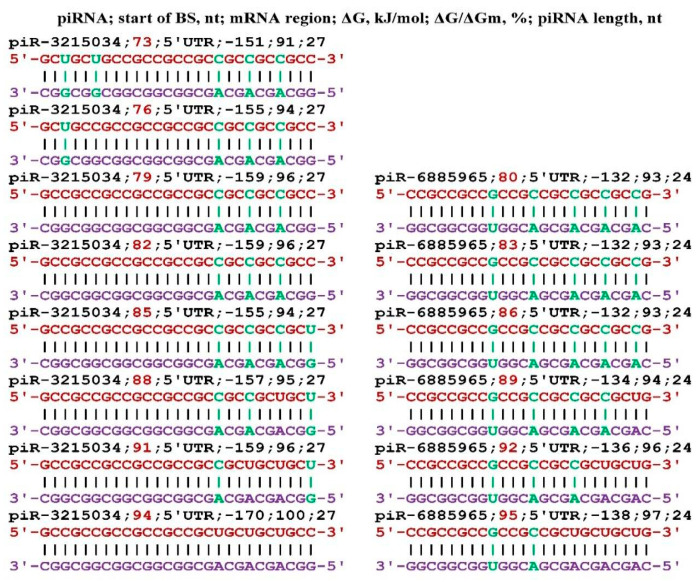
Schemes of the interaction of piR-3215034 and piR-6885965 with mRNA of the *WBP4* gene.

The *WBP4* gene (synonymous name *FBP21*) is involved in splicing and therefore affects the maturation of the mRNA of many genes involved in metabolism [74]. Eight piR-3215034 and six piR-6885965 bind to the mRNA of the *WBP4* gene (Figure 9). At position 94 nt, the 5′UTR of piR-3215034 binds only via canonical base pairs. piR-6885965 binds at the 94 nt position, also with a high ΔG/ΔGm value of 97%. No other piRNAs interacting with mRNA of the *WBP4* gene were identified.

**Figure 10:**
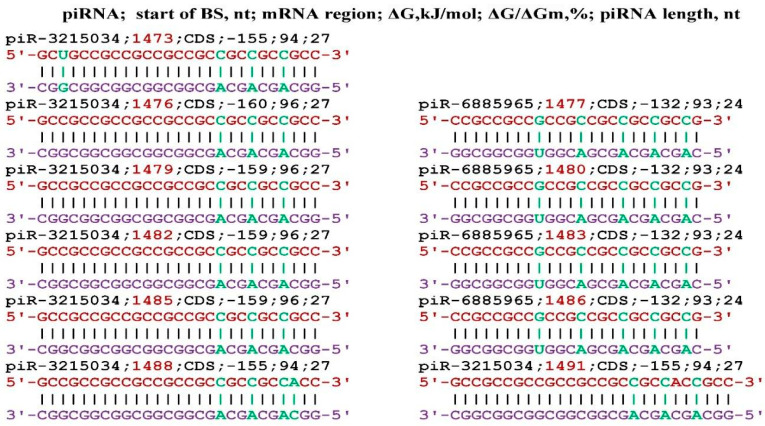
Schemes of the interaction of piR-3215034 and piR-6885965 with the mRNA of the *ZIC5* gene.

The *ZIC5* gene acts as a transcriptional repressor. Increased expression of this gene is observed in various types of human cancer and may contribute to cancer progression [75,76,77]. Figure 10 shows the interaction schemes of piR-3215034 and piR-6885965 with the mRNA of the *ZIC5* gene, which show a high degree of influence of piR-3215034 on translation. At four positions, the ΔG value is −159 kJ/mol and the ΔG/ΔGm ratio is 96% of the maximum value. For piR-6885965, there are only four BSs in the cluster with a ΔG/ΔGm value greater than 90%. No other piRNAs interacting with mRNA of the *ZIC5* gene were identified.

**Figure 11:**
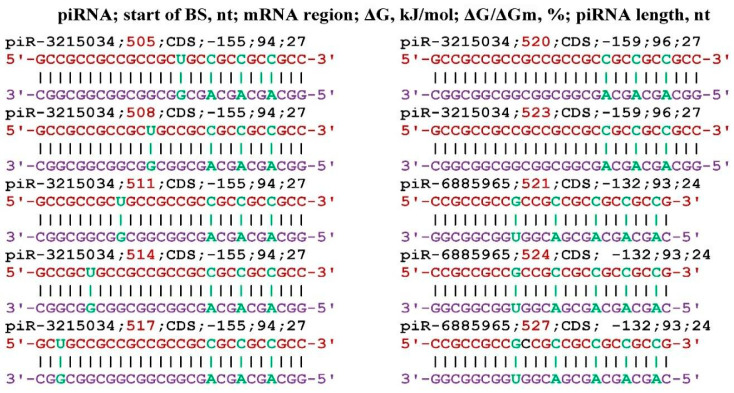
Schemes of interaction of piR-3215034 and piR-6885965 with the mRNA of the *ZSWIM6* gene.

The transcription factor encoded by the *ZSWIM6* gene is synthesized in the brain and can affect the expression of a number of genes. Mutations in this gene lead to malformations of the brain [78,79]. Figure 11 shows the binding schemes of piR-3215034 and piR-6885965 to the mRNA of the *ZSWIM6* gene. piR-3215034 had seven BSs forming a cluster and piR-6885965 had only three BSs in the same cluster. Transcription factors are difficult to study because the product of their activity can be diverse and difficult to control. The importance of their biological role is undoubted. No other piRNAs interacting with mRNA of the *ZSWIM6* gene were identified.

**Figure 12:**
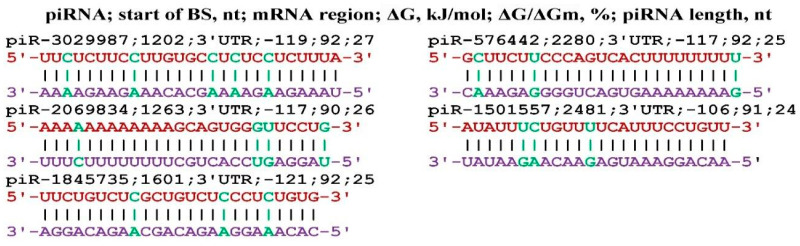
Schemes of piRNA interaction with the mRNA of the *FGF23* gene.

The *FGF23* gene is a member of a large family of fibroblast growth factors [80], which is most associated with the *KL* gene in anti-aging processes. A number of publications have examined the relationship of *FGF23* and *KL* genes in physiological processes and in various diseases [81,82]. In this regard, we studied the possible effect of piRNA on the expression of the *FGF23* gene. Only piR-576442, piR-1501557, piR-1845735, piR-2069834, and piR-3029987 could bind to the mRNA of the *FGF23* gene, the interaction schemes of which are shown in Figure 12. All BSs of these piRNAs were located in the 3′UTR at a considerable distance from each other, i.e., BSs did not form BS clusters.

## 4. Discussion

We have shown that some piRNAs are capable of suppressing *KL* synthesis and can suppress the expression of other genes involved in the development of various diseases. Therefore, if the concentration of these piRNAs is reduced, *KL* expression may increase, while the expression of these disease-related genes may also change. To increase lifespan, selective reduction in piRNAs that specifically inhibit *KL* expression is required. Some authors call Klotho protein a hormone, others an anti-inflammatory agent. The latter function has reason to be because the composition of Klotho protein has an increased content of phenylalanine compared to conventional proteins and is comparable to antioxidant proteins.

Common piRNAs for several genes represent a pool of gene expression regulators and maintain the expression homeostasis of these target genes. A change in the expression of any of these genes will cause a redistribution of the degree of influence of the piRNAs on other genes. Therefore, to increase longevity it is necessary to reduce the concentration of only piRNA-6890096 which suppresses the expression of the *KL* gene with high selectivity. Some publications have identified an anti-inflammatory effect of the Klotho protein. The latter function has reason to be because the Klotho protein has an increased phenylalanine content (6%) compared to the phenylalanine content of conventional proteins and is comparable to antioxidant proteins.

To increase lifespan, it is necessary to reduce the concentration of those piRNAs that suppress the expression of only the *KL* gene with high selectivity. Such a piRNA is piRNA-6890096, which binds completely complementarily to the mRNA of the gene and practically does not affect the expression of other human genes. One way to selectively reduce the concentration of piRNA-6890096 is to introduce sponging RNA containing the binding sites of this piRNA. Such sponging of mRNAs in the annular conformation can reduce free piRNA-6890096. Many publications have shown that sponging RNAs effectively reduces the effect of mRNA. Of the 7310 miRNAs, only ID00756.3p-miR can bind to the mRNA of the *KL* gene. Consequently, piRNA can also be synthesized for it, which will specifically reduce the concentration in blood and cells. The piRNAs identified in this work that bind to mRNAs of protein-coding genes form associations of piRNAs and the target gene, which reflect a specific relationship between several piRNAs and genes. Such associations of piRNA and the target gene, depending on a particular disease, change in different ways. Therefore, such associations can be used as a diagnostic test. A decrease or increase in the concentration of piRNA, due to the high specificity of associations, will unequivocally indicate a disease. The obtained results indicate that it is possible to identify associations of piRNA and miRNA with the *KL* and *FGF23* genes, which will allow for the regulation of their expression. To avoid the side effects of these piRNAs and miRNAs, it is proposed to use sponging RNAs capable of highly selectively binding to piRNAs and miRNAs that suppress *KL* and *FGF23* gene expression.

The results of this study demonstrate that identifying the interaction of a single miRNA with the mRNA of a single gene does not ensure that this miRNA can be used to regulate only that gene. A given miRNA may regulate the expression of multiple genes, potentially leading to unintended or adverse effects. For example, an miRNA may suppress the expression of an oncogene while simultaneously inhibiting a tumor suppressor gene involved in another disease. Conversely, suppression of a tumor suppressor gene may promote the expression of other oncogenes or disease-associated genes. In this way, miRNAs can influence a wide range of physiological processes.

Therefore, it is essential to determine the effects of a specific miRNA on the expression of all genes within an organism. A comprehensive assessment of miRNA function requires evaluating its impact across the entire set of protein-coding genes. However, conducting such large-scale analyses using experimental (wet-lab) methods is costly and time-consuming. Computational approaches enable the systematic identification of miRNA interactions with all genes in an organism, significantly reducing both time and cost. Moreover, computational methods enable the quantitative characterization of miRNA interactions with specific mRNAs, providing more detailed insight into the interaction properties of different miRNA–mRNA pairs.

The mechanisms underlying piRNA–mRNA interactions are similar to those of miRNAs. More than eight million piRNAs are present in the human genome, which dramatically increases the complexity, time, and cost of experimentally characterizing their interactions with approximately one hundred thousand human gene isoforms. This challenge largely explains the limited current understanding of piRNA-mediated gene regulation. Evidence indicates that miRNA and piRNA expression levels change during ontogenesis, suggesting that these small RNAs play genome-wide regulatory roles in animals, including humans. Consequently, elucidating the global influence of miRNAs and piRNAs on human gene expression represents a major objective in modern biomedical research.

## 5. Conclusions

This computational analysis identified predicted interactions between piRNAs and the mRNAs of protein-coding genes, forming specific piRNA-target gene associations. These interactions suggest selective associations between multiple piRNAs and their predicted target genes, which may vary depending on the specific disease. Consequently, the identified interaction patterns represent potential candidates for further investigation in diagnostic research. Changes in piRNA abundance, together with the specificity of their predicted target interactions, may serve as molecular indicators of disease; however, these findings require validation through experimental and clinical studies.

## Data Availability

All relevant data are presented within the paper.

## References

[1] Kuro-o M., Matsumura Y., Aizawa H., Kawaguchi H., Suga T., Utsugi T., Ohyama Y., Kurabayashi M., Kaname T., Kume E. (1997). Mutation of the Mouse Klotho Gene Leads to a Syndrome Resembling Ageing. Nature.

[2] Li S.-S., Sheng M., Sun Z.-Y., Liang Y., Yu L.-X., Liu Q.-F. (2023). Upstream and Downstream Regulators of Klotho Expression in Chronic Kidney Disease. Metabolism.

[3] Ni W., Zhang Y., Yin Z. (2021). The Protective Mechanism of Klotho Gene-Modified Bone Marrow Mesenchymal Stem Cells on Acute Kidney Injury Induced by Rhabdomyolysis. Regen. Ther..

[4] Jiang Y., Jiang W., Li Y., Gu W., Huang H., Wei Q., Bai G., Wang J., Rizak J.D., Zhou Z. (2022). Evaluation of Klotho Gene Expression and NGAL Levels Following Acute Kidney Injury during Pregnancy Hypertensive Disorders. Pregnancy Hypertens.

[5] Xia J., Cao W. (2021). Epigenetic Modifications of Klotho Expression in Kidney Diseases. J. Mol. Med..

[6] Iijima H., Gilmer G., Wang K., Bean A.C., He Y., Lin H., Tang W.-Y., Lamont D., Tai C., Ito A. (2023). Age-Related Matrix Stiffening Epigenetically Regulates α-Klotho Expression and Compromises Chondrocyte Integrity. Nat. Commun..

[7] Aziz M.S., Aamir A.-H., Khan A., Khan Z., Shah S.Q., Safi S.Z., Batumalaie K., Alobaid H.M., Ali A., Imran M. (2022). Investigation of Klotho G395A and C1818T Polymorphisms and Their Association with Serum Glucose Level and Risk of Type 2 Diabetes Mellitus. Genes.

[8] Speer T., Schunk S.J. (2022). Klotho in Diabetic Kidney Disease: More than Dust in the Wnt. Kidney Int..

[9] Gong Z., Banchs P.A.P., Liu Y., Fu H., Arena V.C., Forno E., Libman I., Ho J., Muzumdar R. (2022). Serum α-KL, a Potential Early Marker of Diabetes Complications in Youth with T1D, Is Regulated by miRNA 192. Front. Endocrinol..

[10] Biscetti F., Rando M.M., Cecchini A.L., Nicolazzi M.A., Rossini E., Angelini F., Iezzi R., Eraso L.H., Dimuzio P.J., Pitocco D. (2023). The Role of Klotho and FGF23 in Cardiovascular Outcomes of Diabetic Patients with Chronic Limb Threatening Ischemia: A Prospective Study. Sci. Rep..

[11] Typiak M., Piwkowska A. (2021). Antiinflammatory Actions of Klotho: Implications for Therapy of Diabetic Nephropathy. Int. J. Mol. Sci..

[12] Wang M., Zhang J., Kalantar-Zadeh K., Chen J. (2023). Focusing on Phosphorus Loads: From Healthy People to Chronic Kidney Disease. Nutrients.

[13] Donate-Correa J., Matos-Perdomo E., González-Luis A., Martín-Olivera A., Ortiz A., Mora-Fernández C., Navarro-González J.F. (2023). The Value of Klotho in Kidney Transplantation. Transplantation.

[14] Kim H.J., Kim Y., Kang M., Kim S., Park S.K., Sung S., Hyun Y.Y., Jung J.Y., Ahn C., Oh K.-H. (2022). Low Klotho/Fibroblast Growth Factor 23 Ratio Is an Independent Risk Factor for Renal Progression in Chronic Kidney Disease: Finding From KNOW-CKD. Front. Med..

[15] Liu S., Yao W. (2022). Prediction of Lung Cancer Using Gene Expression and Deep Learning with KL Divergence Gene Selection. BMC Bioinform..

[16] Xie B., Chen J., Liu B., Zhan J. (2013). Klotho Acts as a Tumor Suppressor in Cancers. Pathol. Oncol. Res..

[17] Kim G., Chung H., Lee S., Kim W.H. (2023). Reduced Klotho Expression and Its Prognostic Significance in Canine Hepatocellular Carcinoma. Vet. Comp. Oncol..

[18] Chung H., Lee S., Kim G.A., Kim W.H. (2022). Down-Expression of Klotho in Canine Mammary Gland Tumors and Its Prognostic Significance. PLoS ONE.

[19] Terzi Demirsoy E., Mehtap O., Birtas Atesoglu E., Tarkun P., Gedük A., Eren N., Hacihanefioglu A. (2022). Prognostic Value of Serum Soluble Klotho and Fibroblast Growth Factor-23 in Multiple Myeloma Patients. Indian J. Hematol. Blood Transfus..

[20] Saxena A., Sachan T., Gupta A., Kapoor V. (2022). Effect of Dietary Phosphorous Restriction on Fibroblast Growth 2 Factor-23 and sKlotho Levels in Patients with Stages 1–2 Chronic Kidney Disease. Nutrients.

[21] Mizuno Y., Ishida T., Kugimiya F., Takai S., Nakayama Y., Yonemitsu K., Harada E. (2023). Deterioration of Phosphate Homeostasis Is a Trigger for Cardiac Afterload ― Clinical Importance of Fibroblast Growth Factor 23 for Accelerated Aging. Circ. Rep..

[22] Liu S.-H., Xiao Z., Mishra S.K., Mitchell J.C., Smith J.C., Quarles L.D., Petridis L. (2022). Identification of Small-Molecule Inhibitors of Fibroblast Growth Factor 23 Signaling via In Silico Hot Spot Prediction and Molecular Docking to α-Klotho. J. Chem. Inf. Model..

[23] Yanucil C., Kentrup D., Campos I., Czaya B., Heitman K., Westbrook D., Osis G., Grabner A., Wende A.R., Vallejo J. (2022). Soluble α-Klotho and Heparin Modulate the Pathologic Cardiac Actions of Fibroblast Growth Factor 23 in Chronic Kidney Disease. Kidney Int..

[24] Abraham C.R., Li A. (2022). Aging-Suppressor Klotho: Prospects in Diagnostics and Therapeutics. Ageing Res. Rev..

[25] Prud’homme G.J., Kurt M., Wang Q. (2022). Pathobiology of the Klotho Antiaging Protein and Therapeutic Considerations. Front. Aging.

[26] Orces C.H. (2022). The Association between Metabolic Syndrome and the Anti-Aging Humoral Factor Klotho in Middle-Aged and Older Adults. Diabetes Metab. Syndr. Clin. Res. Rev..

[27] Liu Y., Bi X., Xiong J., Han W., Xiao T., Xu X., Yang K., Liu C., Jiang W., He T. (2019). MicroRNA-34a Promotes Renal Fibrosis by Downregulation of Klotho in Tubular Epithelial Cells. Mol. Ther..

[28] Alsenousy A.H.A., Sharker S.A., Gowayed M.A., Elblehi S.S., Kamel M.A. (2025). Aptamer-Targeted Anti-miR RNA Construct Based on 3WJ as a New Approach for the Treatment of Chronic Kidney Disease in an Experimental Model. Gene Ther..

[29] Yadav D., Birdi A., Tomo S., Nebhinani N., Sharma P. (2023). Association of Klotho Gene Expression and miRNA- 339 in Schizophrenia. Eur. Psychiatry.

[30] Zhang D., Li Z., Gao Y., Sun H. (2025). MiR-556-3p Mediated Repression of Klotho under Oxidative Stress Promotes Fibrosis of Renal Tubular Epithelial Cells. Sci. Rep..

[31] Morii K., Yamasaki S., Doi S., Irifuku T., Sasaki K., Doi T., Nakashima A., Arihiro K., Masaki T. (2019). microRNA-200c Regulates KLOTHO Expression in Human Kidney Cells under Oxidative Stress. PLoS ONE.

[32] Chen K., Zhang B., Sun Z. (2021). MicroRNA 379 Regulates Klotho Deficiency-Induced Cardiomyocyte Apoptosis Via Repression of Smurf1. Hypertension.

[33] Zhang X., Li L., Tan H., Hong X., Yuan Q., Hou F.F., Zhou L., Liu Y. (2024). Klotho-Derived Peptide 1 Inhibits Cellular Senescence in the Fibrotic Kidney by Restoring Klotho Expression via Posttranscriptional Regulation. Theranostics.

[34] Belkozhayev A., Niyazova R., Wilson C., Jainakbayev N., Pyrkova A., Ashirbekov Y., Akimniyazova A., Sharipov K., Ivashchenko A. (2022). Bioinformatics Analysis of the Interaction of miRNAs and piRNAs with Human mRNA Genes Having Di- and Trinucleotide Repeats. Genes.

[35] Kamenova S., Sharapkhanova A., Akimniyazova A., Kuzhybayeva K., Kondybayeva A., Rakhmetullina A., Pyrkova A., Ivashchenko A. (2022). piRNA and miRNA Can Suppress the Expression of Multiple Sclerosis Candidate Genes. Nanomaterials.

[36] Wang J., Shi Y., Zhou H., Zhang P., Song T., Ying Z., Yu H., Li Y., Zhao Y., Zeng X. (2022). piRBase: Integrating piRNA Annotation in All Aspects. Nucleic Acids Res..

[37] Londin E., Loher P., Telonis A.G., Quann K., Clark P., Jing Y., Hatzimichael E., Kirino Y., Honda S., Lally M. (2015). Analysis of 13 Cell Types Reveals Evidence for the Expression of Numerous Novel Primate- and Tissue-Specific microRNAs. Proc. Natl. Acad. Sci. USA.

[38] Backes C., Meder B., Hart M., Ludwig N., Leidinger P., Vogel B., Galata V., Roth P., Menegatti J., Grässer F. (2016). Prioritizing and Selecting Likely Novel miRNAs from NGS Data. Nucleic Acids Res..

[39] Ivashchenko A., Berillo O., Pyrkova A., Niyazova R., Atambayeva S. (2014). MiR-3960 Binding Sites with mRNA of Human Genes. Bioinformation.

[40] Garg A., Heinemann U. (2018). A Novel Form of RNA Double Helix Based on G·U and C·A ^+^ Wobble Base Pairing. RNA.

[41] Leontis N.B. (2002). The Non-Watson-Crick Base Pairs and Their Associated Isostericity Matrices. Nucleic Acids Res..

[42] Fire A., Xu S., Montgomery M.K., Kostas S.A., Driver S.E., Mello C.C. (1998). Potent and Specific Genetic Interference by Double-Stranded RNA in Caenorhabditis Elegans. Nature.

[43] Atambayeva S., Niyazova R., Ivashchenko A., Pyrkova A., Pinsky I., Akimniyazova A., Labeit S. (2017). The Binding Sites of miR-619-5p in the mRNAs of Human and Orthologous Genes. BMC Genom..

[44] Kuo Y.-J., Lewis J.S., Truong T., Yeh Y.-C., Chernock R.D., Zhai C., Chen Y.-A., Hongo T., Lee C.-K., Shi Q. (2022). Nuclear Expression of AFF2 C-Terminus Is a Sensitive and Specific Ancillary Marker for DEK::AFF2 Carcinoma of the Sinonasal Tract. Mod. Pathol..

[45] Savari O., Chang J.C., Bishop J.A., Sakthivel M.K., Askin F.B., Rekhtman N. (2022). First Report of Thoracic Carcinoma With DEK::AFF2 Rearrangement: A Case Report. J. Thorac. Oncol..

[46] Ruangritchankul K., Sandison A. (2023). DEK::AFF2 Fusion Carcinomas of Head and Neck. Adv. Anat. Pathol..

[47] Ji Z., Lu R., Wu T., Chen Z., Wen Z., Li Z., Zheng X., Tang J., Chen X., Yang Y. (2023). Expression Profiling of Circular RNA Reveals a Potential miR-145-5p Sponge Function of Circ-AFF2 and Circ-ASAP1 in Renal Cell Carcinoma. Am. J. Transl. Res..

[48] Taherkhani A., Dehto S.S., Jamshidi S., Shojaei S. (2022). Pathogenesis and Prognosis of Primary Oral Squamous Cell Carcinoma Based on microRNAs Target Genes: A Systems Biology Approach. Genom. Inf..

[49] Flores D., Lopez A., Udawant S., Gunn B., Keniry M. (2023). The FOXO1 Inhibitor AS1842856 Triggers Apoptosis in Glioblastoma Multiforme and Basal-like Breast Cancer Cells. FEBS Open Bio.

[50] Rose M.M., Espinoza V.L., Hoff K.J., Pike L.A., Sharma V., Hofmann M.-C., Tan A.C., Pozdeyev N., Schweppe R.E. (2023). BCL2L11 Induction Mediates Sensitivity to Src and MEK1/2 Inhibition in Thyroid Cancer. Cancers.

[51] Ranapour S., Motamed N. (2023). Effect of Silibinin on the Expression of Mir-20b, Bcl2L11, and Erbb2 in Breast Cancer Cell Lines. Mol. Biotechnol..

[52] Li J., Zheng W., Wu J., Zhang J., Lv B., Li W., Liu J., Zhang X., Huang T., Luo Z. (2023). CPT1C-Mediated Fatty Acid Oxidation Facilitates Colorectal Cancer Cell Proliferation and Metastasis. Acta Biochim. Biophys. Sin..

[53] Liao L., Zhang F., Zhuo Z., Huang C., Zhang X., Liu R., Gao B., Ding S. (2023). Regulation of Fatty Acid Metabolism and Inhibition of Colorectal Cancer Progression by Erchen Decoction. Evid.-Based Complement. Altern. Med..

[54] Xiong L., He T., Liu C., Qin S., Xiao T., Xin W., Wang Y., Ran L., Zhang B., Zhao J. (2023). IL-37 Ameliorates Renal Fibrosis by Restoring CPT1A-Mediated Fatty Acid Oxidation in Diabetic Kidney Disease. Kidney Dis..

[55] Li S., Liu M., Chen J., Chen Y., Yin M., Zhou Y., Li Q., Xu F., Li Y., Yan X. (2023). L-Carnitine Alleviates Cardiac Microvascular Dysfunction in Diabetic Cardiomyopathy by Enhancing PINK1-Parkin-Dependent Mitophagy through the CPT1a-PHB2-PARL Pathways. Acta Physiol..

[56] Tian T., Lu Y., Lin J., Chen M., Qiu H., Zhu W., Sun H., Huang J., Yang H., Deng W. (2022). CPT1A Promotes Anoikis Resistance in Esophageal Squamous Cell Carcinoma via Redox Homeostasis. Redox Biol..

[57] Bernard J.N., Chinnaiyan V., Andl T., Le Bras G.F., Qureshi M.N., Altomare D.A., Andl C.D. (2022). Augmented CPT1A Expression Is Associated with Proliferation and Colony Formation during Barrett’s Tumorigenesis. Int. J. Mol. Sci..

[58] Deng J.J., Li G.P., Lu W., Yan Z., Wang Y. (2022). DAZAP1 Overexpression Promotes Growth of HCC Cell Lines: A Primary Study Using CEUS. Clin. Transl. Oncol..

[59] Wang Q., Guo Y., Wang W., Liu B., Yang G., Xu Z., Li J., Liu Z. (2021). RNA Binding Protein DAZAP1 Promotes HCC Progression and Regulates Ferroptosis by Interacting with SLC7A11 mRNA. Exp. Cell Res..

[60] Kim M.C., Park M.H., Kang S.H., Bae Y.K. (2019). NDRG3 Protein Expression Is Associated with Aggressive Biologic Phenotype and Unfavorable Outcome in Patients with Invasive Breast Cancer. Int. J. Clin. Exp. Pathol..

[61] Liu Y., Xia J., Zhou Y., Shao S. (2021). High Expression of NDRG3 Correlates with Poor Prognosis in Gastric Cancer Patients. Rev. Esp. Enferm. Dig..

[62] Ma W., Zhao X., Xue N., Gao Y., Xu Q. (2021). The LINC01410/miR-122-5p/NDRG3 Axis Is Involved in the Proliferation and Migration of Osteosarcoma Cells. IUBMB Life.

[63] Pappula A.L., Rasheed S., Mirzaei G., Petreaca R.C., Bouley R.A. (2021). A Genome-Wide Profiling of Glioma Patients with an IDH1 Mutation Using the Catalogue of Somatic Mutations in Cancer Database. Cancers.

[64] Yin X., Yu H., He X.-K., Yan S.-X. (2022). Prognostic and Biological Role of the N-Myc Downstream-Regulated Gene Family in Hepatocellular Carcinoma. World J. Clin. Cases.

[65] Wang J., Wang J., Quan J., Liu J., Tian L., Dong C. (2022). Relationship between Serum NDRG3 and Papillary Thyroid Carcinoma. Front. Endocrinol..

[66] Zhang H., Ge Z., Wang Z., Gao Y., Wang Y., Qu X. (2021). Circular RNA RHOT1 Promotes Progression and Inhibits Ferroptosis via Mir-106a-5p/STAT3 Axis in Breast Cancer. Aging.

[67] Li Q., Yao L., Wei Y., Geng S., He C., Jiang H. (2015). Role of RHOT1 on Migration and Proliferation of Pancreatic Cancer. Am. J. Cancer Res..

[68] Jiang H., He C., Geng S., Sheng H., Shen X., Zhang X., Li H., Zhu S., Chen X., Yang C. (2012). RhoT1 and Smad4 Are Correlated with Lymph Node Metastasis and Overall Survival in Pancreatic Cancer. PLoS ONE.

[69] Wang L., Long H., Zheng Q., Bo X., Xiao X., Li B. (2019). Circular RNA circRHOT1 Promotes Hepatocellular Carcinoma Progression by Initiation of NR2F6 Expression. Mol. Cancer.

[70] Periñán M.T., Gómez-Garre P., Blauwendraat C., Mir P., Bandres-Ciga S., International Parkinson’s Disease Genomics Consortium (IPDGC) (2021). The Role of RHOT1 and RHOT2 Genetic Variation on Parkinson Disease Risk and Onset. Neurobiol. Aging.

[71] Zehtabcheh S., Soleimani Samarkhazan H., Asadi M., Zabihi M., Parkhideh S., Mohammadi M.H. (2025). Insights into KMT2A Rearrangements in Acute Myeloid Leukemia: From Molecular Characteristics to Targeted Therapies. Biomark. Res..

[72] Gulisija D., Gonzalez-Reymundez A., Fenton J.I., de Los Campos G., Bray M.S., Vazquez A.I. (2025). Uncovering Covariance Patterns across Energy Balance Traits Enables the Discovery of New Obesity-Related Genes. Obesity.

[73] Tamai M., Komatsu C., Kagami K., Kasai S., Watanabe A., Akahane K., Goi K., Tomoyasu C., Imamura T., Oguri S. (2025). A Characteristic Gene Expression Profile Regulated by ACIN1::NUTM1 Fusion in a Newly Identified Infant Leukaemic Cell Line and an ACIN1::NUTM1-Inducible Model. Br. J. Haematol..

[74] Xing Q., Liu S., Luan J., Wang Y., Ma L. (2021). A Novel 13 RNA Binding Proteins (RBPs) Signature Could Predict Prostate Cancer Biochemical Recurrence. Pathol. Res. Pract..

[75] Jia Q., Song J., Xu T., Liu J., Chai J., Yang Y., Li L., Li M., Yang X. (2023). ZIC5 Promotes Aggressiveness and Cancer Stemness in Cervical Squamous Cell Carcinoma. Pathol. Res. Pract..

[76] Tan Y.-F., Zhang Y., Ge S.-Y., Zhong F., Sun C.-Y., Xia G.-W. (2022). AR-Regulated ZIC5 Contributes to the Aggressiveness of Prostate Cancer. Cell Death Discov..

[77] Song W., Yu W., Li D., Cheng C., Wu X., Chen J., Zhang W. (2022). ZIC5 Promotes Human Hepatocellular Carcinoma Cell Proliferation through Upregulating COL1A1. J. Gastrointest. Oncol..

[78] Yanagishita T., Eto K., Yamamoto-Shimojima K., Segawa O., Nagata M., Ishihara Y., Miyashita Y., Asano Y., Sakata Y., Nagata S. (2021). A Recurrent de Novo ZSWIM6 Variant in a Japanese Patient with Severe Neurodevelopmental Delay and Frequent Vomiting. Hum. Genome Var..

[79] Tischfield D.J., Saraswat D.K., Furash A., Fowler S.C., Fuccillo M.V., Anderson S.A. (2017). Loss of the Neurodevelopmental Gene Zswim6 Alters Striatal Morphology and Motor Regulation. Neurobiol. Dis..

[80] Kubota M., Hamasaki Y., Hashimoto J., Aoki Y., Kawamura T., Saito A., Yuasa R., Muramatsu M., Komaba H., Toyoda M. (2023). Fibroblast Growth Factor 23-Klotho and Mineral Metabolism in the First Year after Pediatric Kidney Transplantation: A Single-center Prospective Study. Pediatr. Transplant..

[81] Balcázar-Hernández L., Manuel-Apolinar L., Vargas Ortega G., González-Virla B., Reza-Albarrán A.A., Martínez Jiménez M.D.C., Martínez Ordaz J.L., Mendoza-Zubieta V., Basurto L. (2023). Vitamin D and Its Positive Effect on the PTH/Vitamin D/Calcium-FGF23/Klotho/Phosphorus Axis in Kidney Transplant Recipients. Nutr. Hosp..

[82] Nakano T., Kishimoto H., Tokumoto M. (2023). Direct and Indirect Effects of Fibroblast Growth Factor 23 on the Heart. Front. Endocrinol..

